# Current trends in treatment of hypertension in Karachi and cost minimization possibilities

**DOI:** 10.12669/pjms.315.7438

**Published:** 2015

**Authors:** Izhar M. Hussain, Baqir S. Naqvi, Rao M. Qasim, Nasir Ali

**Affiliations:** 1 Izhar M. Hussain, M. Pharm., MBA. Director, Center for Executive Education, Institute of Business Administration, IBA City Campus, Garden / Kiyani Shaheed Road, Karachi, Pakistan; 2 Dr. Baqir S. Naqvi, PhD. Professor, Department of Pharmaceutics, Faculty of Pharmacy, Hamdard University, Karachi, Pakistan; 3 Rao M. Qasim, Pharm. D, M. Phil., MBA Institute of Business Management, Karachi, Pakistan; 4 Nasir Ali, MBA Supply Chain Management, Research Executive, IBA Center for Executive Education, Karachi, Pakistan

**Keywords:** Cost minimization, Stage I hypertension, Initial therapy, Evidence-based therapeutic guidelines, Compelling indications

## Abstract

**Objective:**

This study finds out drug usage trends in Stage I Hypertensive Patients without any compelling indications in Karachi, deviations of current practices from evidence based antihypertensive therapeutic guidelines and looks for cost minimization opportunities.

**Methods:**

In the present study conducted during June 2012 to August 2012, two sets were used. Randomized stratified independent surveys were conducted in doctors and general population - including patients, using pretested questionnaires. Sample sizes for doctors and general population were 100 and 400 respectively. Statistical analysis was conducted on Statistical Package for Social Science (SPSS). Financial impact was also analyzed.

**Results:**

On the basis of patients’ doctors’ feedback, Beta Blockers, and Angiotensin Converting Enzyme Inhibitors were used more frequently than other drugs. Thiazides and low-priced generics were hardly prescribed. Beta blockers were prescribed widely and considered cost effective. This trend increases cost by two to ten times.

**Conclusion:**

Feedbacks showed that therapeutic guidelines were not followed by the doctors practicing in the community and hospitals in Karachi. Thiazide diuretics were hardly used. Beta blockers were widely prescribed. High priced market leaders or expensive branded generics were commonly prescribed. Therefore, there are great opportunities for cost minimization by using evidence-based clinically effective and safe medicines.

## INTRODUCTION

Hypertension is the major risk factor for cardiovascular disease. The WHO declared it ‘the number one killer’ in The World Health Report of year 2001. It is important to note that hypertensive patients have four times greater chances for stroke and Two times greater chances myocardial infarction (a heart attack) than those who have normal blood pressure.^1^ The chances of complications increase with rise in level of blood pressure. It is reported that a 5mmHg drop of blood pressure, reduces risk of stroke by 14%, coronary heart disease by 9% and overall chances of mortality by 7%.

Hypertension is the most common cardiovascular disease in Pakistan as well. The National Heath Survey of Pakistan reported incidence of high blood pressure in 18% of adults over 18 year of age and 33% of adults over 45 years. There are different reports showing different figures of rates of prevalence, awareness, treatment and control. The experts developed guidelines for safe and better management of hypertension for the health providers and general public on the basis of clinically and scientifically sound and validated evidences. Juxtaposing, different guidelines (Table-I) for initial or Stage I therapy for hypertension without compelling indications use of Thiazide Diuretics, Angiotensin Converting Enzyme Inhibitors (ACEIs) or Calcium Channel Blockers (CCB) are widely recommended. The JNCVII Guidelines strongly recommends thiazide for “most of the patients”. Pakistan Hypertension Leagues’ guideline has more or less adopted NICE (UK) recommendations. NICE recommends ACEIs to patients younger than 55years whereas CCB and diuretics to patients 55 years or blacks. Beta Blockers (BBs), once considered as key medication, are not recommended for initial or Stage I therapy for hypertension except in compelling conditions. Combinations of more than one drug are also encouraged to achieve therapeutic goal. Use of generic drugs is recommended to reduce prescription cost.

**Table-I:** Summary of National and International Guidelines for the Management of Hypertension.

S. No.	Excerpts from the Guidelines	Evidence-based clinically ascertained guidelines for the management of hypertension				
		PHL^13^	JNC VII^14^	WHO/ISH^15^	NICE^16^	South Africa^17^
1	Initial Drug Choice:	Step 1: Younger than 55 years and non-black: ACEIs & older than 55 years or black: CCBs & Diuretics	Thiazides for most of the patients	ACEIs, CCBs & Diuretics	Step 1: Younger than 55 years and non-black: ACEIs & older than 55 years or black: CCB & Diuretic	For the majority of patients without a compelling indication for another class of drug. A low cost diuretic should be considered as first choice therapy on the basis of comparative trial data, availability and cost.
2	Cost reducing measures:	“Diuretics, being very economic drugs may be used as a first line therapy in countries like Pakistan,.”	Recommended	Recommended	Recommended	A thiazide-like diuretic is advisable when consideration for cost of therapy is relevant.
3	Use of BBs for initial therapy:	More recently the BBs are deemphasized due to their diabetogenic potentials.	Not recommended	Not recommended	BBs not to be preferred for initial hypertension therapy (except for compelling condition).	Not recommended

***Abbreviations:*** PHL = Pakistan Hypertension League. JNC = Joint National Commission on Hypertension, National Institute of Health USA. WHO = World Health Organization. ISH = International Society of Hypertension. NICE = National Institute Health, UK. ACEIs = Angiotensin Converting Enzyme Inhibitors. CCBs = Calcium Channel Blockers. BBs = Beta Blockers.

Key issues, globally, are lack of adherence to therapeutic guidelines for hypertensive therapy by the doctors, and lack of patients’ awareness of disease as well as compliance to their doctors’ recommendations. In Pakistan, the situation is not different; there are reports of very low awareness of disease and observance to doctors’ advicein patients and wide deviations to the therapeutic guidelines available for health providers in Pakistan. Jafar et al. (2005) asked for special efforts to encourage doctors (particularly general practitioners) for prescribing cost effective regimen.^2^ In another study by Hameed et al. (2004) reported misconceptions in understanding and treatment of hypertension among a group of doctors. He found that 50% of the general practitioners (GPs) attending a Continuous Medical Education (CME) workshop on hypertension could not define hypertension, whereas 75% of them believed that anxiolytics were the first line therapy for hypertension.^3^ The major hurdles in the management of hypertension that emerged from the reports were financial constraints, non-compliance to treatment regime and lack of follow-up with physicians.^4^

Impact of hypertension on healthcare cost is huge. In USA, it cost US$ 73.4 billion in the year 2009. The financial requirements for antihypertensive therapy in Brazil was US$ 9.6 billion in 2012 with 24% increase during the year 2010 -2012.^5^,^6^ Search and efforts for reducing cost of antihypertensive therapy has been intensive worldwide i.e. Brazil, Caribbean countries, Malaysia, Poland, and USA.^7^-^10^ It was found by the American Heart Association (ASA) that the medication cost was 45% of total direct cost of therapy.^11^ To reduce cost of therapy, Fischer (2004) after studying more than two million prescriptions for antihypertensive medications in 2001, costingUS$ 48.5 million per annum(363 dollars per patient), identified that 40% of prescriptions of medication for which an alternative evidence-based expert recommended cost-effective regimen was available. He calculated that this change would have saved the costs to payers in 2001 by US$11.6 million (nearly a quarter of program spending on antihypertensive medications). Moreover, he suggested replacement was evidence-based for clinical appropriateness on global basis. The largest potential saving was due to replacement of calcium channel blockers.^12^

Our objective was to find out drug usage trends in Stage I Hypertensive Patients without any compelling indications in Karachi, deviations of current practices from evidence based antihypertensive therapeutic guidelines and looking for cost minimization opportunities.

## METHODS

Three pronged approach was used. Two randomized stratified surveys were conducted in health providers (doctors) and health receivers (general population including patients) using pretested questionnaires. Sample size for doctors was 100 (58 general practitioners and 42 doctors working in hospital OPDs) and for general population was 400 (200 males and 200 females) from different socio-economic areas of Karachi. Specialists and consultants were excluded from the study. Data of prescriptions, prescription trends, and drug prices were obtained from authentic sources. Statistical analysis was conducted on Statistical Package for Social Science (SPSS). Financial impact was also analyzed. The study was conducted during June 2012 to August 2012 and it was approved by Ethics Review Committee of Liaquat National Hospital Karachi.

## RESULTS

The present study aims to determine the drug usage trends in Stage I hypertension without any compelling indications in Karachi, identifies deviations of current prescribing practice from the evidence based antihypertensive therapeutic guidelines, and looks for therapeutic cost minimization opportunities.

The Table-II tabulates hypertensive patients’ feedback (N=400) on their first prescribed hypertensive drug. It was found that majority of them were prescribed Beta Blockers (33%), followed by Angiotensin Converting Enzyme (18%), and Calcium Channel Blockers (13%). Diuretics were prescribed to 8% of them. The diuretics included furosemide and amiloride with furosemide. Nobody got thiazide diuretics. Usage of combination drugs was found in 3% of the respondents. It was also found that majority of them got high priced brand leader (original patent). 17% patients could not recall names of their antihypertensive drugs.

**Table-II:** Which drug was given to you when doctor decided to start treating your high blood pressure? (Primary Data – Patients --- N=83).

S. No.	Antihypertensive Class	Score of responses		Main drugs used (name & score)
		Number	%	
1	Beta Blockers (BB)	27	33%	Atenolol # 19 (68% Brand Leader)
				Propranolol # 8
2	Angiotensin Converting Enzyme Inhibitors (ACEIs)	15	18%	Ramipril # 6
				Enalapril # 6 (100% Brand Leader)
3	Calcium Channel Blockers (CCB)	11	13%	Amlodipine # 9
				Nimodipine # 1
				Verapamil # 1
4	Angiotensin Receptor Blockers (ARBs)	7	8%	Losartan # 7 (14% on Brand Leader)
5	Non-Thiazide Diuretics	7	8%	Furosemide # 6 (16% Brand Leader)
				Amiloride with Furosemide # 1
6	Antihypertensive Combinations	2	3%	HCTZ + ACEI # 1
				HCTZ +ARB # 1
7	Thiazide Diuretics (HCTZ)	none	0	No prescription
8	Could not recall drug’s name	14	17%	

Table-III shows doctors response to two queries. When the doctors (N=100) were asked about their choice of first line (Step I) antihypertensive agents in patients with no compelling indications, they preferred Beta Blockers (34%) and ACEIs (34%) over other antihypertensive agents e.g. diuretics (17%), Calcium Channel Blockers (4%), and others (11%). Responding to query asked for their perception of the most cost-effective drug, 29 % of the doctors opined Beta Blockers, followed by Angiotensin Converting Enzymes inhibitors (18%) and diuretics (12%). There was no significant difference in the initial choice of drugs for patients of Stage I hypertension with no compelling indications and perception about cost effectiveness in general practitioners and doctors working in the hospitals.

**Table-III:** Doctors’ first line antihypertensive therapeutic agents and perception of cost effectiveness (Primary Data) [N=100].

S. No.	Classes of Antihypertensive Drugs	Doctors’ choice of drug for initial antihypertensive therapy and perception of cost effectiveness											
		Q: Which is the drug you would like to use as the first line hypertensive in patients without compelling indications?						Q: Which is the most cost effective antihypertensive drug in your opinion?					
		General Practitioners		Hospital Doctors		Overall Total		General Practitioners		Hospital Doctors		Overall Total	
		N	%	N	%	N	%	N	%	N	%	N	%
1	Diuretics	9	16%	8	19%	17	17%	5	9%	7	17%	12	12%
2	Angiotensin Converting Enzymes Inhibitors (ACEIs)	20	34%	14	33%	34	34%	9	16%	9	21%	18	18%
3	Calcium Channel Blockers (CCBs)	3	5%	1	2%	4	4%	3	5%	4	10%	7	7%
4	Angiotensin Receptor Blockers (ARBs)	-	-	-	-	-	-	4	7%	0		4	4%
5	Beta Blockers (BBs)	19	33%	15	36%	34	34%	16	28%	13	31%	29	29%
6	Combinations	-	-	-	-	-	-	7	12%	0	-	7	7%
7	Not mentioned	7	12%	4	10%	11	11%	16	28%	7	17%	22	22%
	Total	58		42		100		58		42		100	

Table-IV gives picture of non-pharmacologic interventions in hypertension mentioned by the patients.84% of the patients acknowledged doctors’ advice for physical exercise whereas 95% remembered doctors’ recommendations for changing dietary habits. Most of the patients recalled medical advice for 30-minute brisk walk for five days a week and salt, fat-rich and high sugar intake reduction.

**Table-IV:** Non-pharmacologic intervention for controlling high blood pressure (Patients’ Feedback)

S. No.	Survey Questions	Subject’s Feedback (N=83)					Additional Feedback related to Survey Questions.
		Yes		No		No Response
		N	%	N	%	%
1	Has your doctor advised you for physical exercise as a measure to control hypertension?	70	84%	12	14%	2%	30-minute brisk walk five days a week
2	Has your doctor or any other health professional ever advised you to change your dietary habits for better control of hypertension?	79	95%	3	4%	1%	•Reduce Salt intake
							•Avoid fatty high sugar containing food.

Table-V gives comparison of annual cost of therapy to a patient. It shows that hydrochlorothiazide(HCTZ) from a national company is the most cost-effective antihypertensive costing only Rs. 367 yearly per patient. When Branded Market Leaders of ACEIs, CCBs, BBs and ARBs annually cost Rs. 3,041; Rs. 6,532; Rs.3,652; and Rs. 28,278 respectively. The Branded Market leaders are 8 to 77 times costlier than above mentioned HCTZ. Even the generic drugs of chemical moieties’ average prices are 5 to 19 times whereas the lowest priced generics are 2 to 8 times more expensive than HCTZ. Price difference between the generics and the branded market leaders ranges from 1.2 to 4 times more expensive. Branded market leaders are 4 to 9 times more expensive than their lowest priced generic drugs.

**Table-V:** Comparison of Annual Cost of Therapy (per Patient).

Antihypertensive Groups	Annual Cost of therapy (Rs)	Other Antihypertensive Vs.HCTZ	Branded Market Leader Vs. Generic product (Average)	Branded Market Leader Vs. Generic product (Lowest price)
*Diuretics : Thiazides*				
Hydrochlorothiazide (HCTZ)	367	1		
[Triamterene:50mg,Hydrochlorothiazide:25mg]	1,243	3		
*Angiotensin Converting Enzyme Inhibitors (ACEIs): Captopril*				
Branded Market Leader	3,041	8		
Generic products (Average)	2,453	7	1.2	
Generic product (Lowest price)	682	2		4
*Calcium Channel Blockers (CCBs): Amlodipine*				
Branded Market Leader	6,532	18		
Generic products (Average)	2,869	8	2.3	
Generic product (Lowest price)	630	2		10
*Beta Blockers (BBs): Atenolol*				
Branded Market Leader	3,652	10		
Generic products (Average)	1,944	5	1.9	
Generic product (Lowest price)	860	2		4
*Angiotensin Receptor Blockers (ARB): Losartan*				
Branded Market Leader	28,278	77		
Generic products (Average)	7,035	19	4.0	
Generic product (Lowest price)	2,984	8		9

## DISCUSSION

Table-I contains relevant points from one national and four international evidence-based therapeutic guidelines for the management and treatment of hypertension. All these guidelines recommend use of thiazide diuretics as the drug of first choice in patients of Stage 1 hypertension without any compelling indications. JNC VII (even JNC VIII) recommends initial therapy with diuretics in such cases. Both the UK’s National Institute of Clinical Excellence (NICE) and Pakistan Hypertension League (PHL) recommend ACEIs for non-black and those younger than 55 years and CCBs and diuretics for black and those older than 55 years. Diuretics are recommended because of safety, cardiovascular protection, and economy. Besides diuretics, ACEIs and CCBs are also recommended. Another important but worth considering point is de-emphasis on use of beta blockers as initial therapy (except for compelling indications). PHL asked for de-emphasize on BBs because of their diabetogenic effects. All these guidelines underscore use of cost effective drugs to minimize cost of therapy.

Feedback received from the patients and the doctors in Karachi showed that non pharmacologic recommendations were given to the patients as per the guidelines. However, gaps were identified in adherence to therapeutic guidelines by the doctors. Deviations in therapy from the guidelines were evident on different counts. For instance, diuretics were not used as recommended and expensive branded leaders were usually prescribed. Beta blockers were extensively used despite strong recommendation by different therapeutic guidelines in hypertensive patients without compelling conditions to deemphasize them because their diabetogenic potential and abstruse cardio protective role.

The perception of cost effectiveness was not clear to doctors. That’s why most of doctors considered Beta Blockers or ACEIs more cost effective than diuretic of thiazide group. Consider a patient taking a thiazide diuretic, would pay Rs. 367 only for a year-long treatment. But if other drugs were used instead of diuretics e.g. beta blockers, ACE Inhibitors, Calcium Channel Blockers or ARBs, yearly medicine cost would go up to Rs. 3,652, Rs. 3,041, Rs.6,532, Rs. 28,278 respectively for corresponding branded market leaders; Rs. 1,944, Rs. 2,452, Rs. 2,869, Rs 7,035 for generic substitutes (average priced) of branded market leaders; and Rs. 860, Rs. 682, Rs. 630, Rs. 2,984 for the lowest priced generic substitutes of branded market leaders registered by the Health Authorities in Pakistan. Because of choice of drugs, cost of therapy could increase many folds ranging from 2 to 77 times depending upon the choice of drugs.

## CONCLUSION

Feedbacks showed that therapeutic guidelines were not followed for choosing pharmacologic agents by the doctors practicing in the community and hospitals in Karachi. Thiazide diuretics were hardly used. Beta blockers were widely prescribed. High priced market leaders or expensive branded generics were commonly prescribed. Therefore, great opportunities for cost minimization by used evidence-based clinically effective and safe medicines. It is concluded that national and international evidence-based therapeutic guidelines for the management of Stage I hypertension in patients without compelling indications were not followed in the selected samples of population and doctors in Karachi. The doctors have misconception about the cost effectiveness of certain antihypertensive agents. The outcomes of this study should be reflected for designing plans for patient awareness and education of health providers; and also considered for periodically updating therapeutic guidelines for blood pressure control.

### Limitations of the study

Some final considerations need elaboration relating to limitations of the study. First, doctors’ viewpoints, for not complying with international therapeutic guidelines, were not explored. Secondly, patients’ opinion about the therapy they were using was also not sought. Moreover, the present work focused in Karachi region only, hence, limiting the targeting population to specific city and could not generalize the results to Pakistan as a whole.
